# Dynamics of an orthopaedic team: Insights to improve teamwork through a design thinking approach

**DOI:** 10.3233/WOR-182777

**Published:** 2018-10-09

**Authors:** E. Caprari, J.T. Porsius, P. D’Olivo, R.M. Bloem, S.B.W. Vehmeijer, N. Stolk, M. Melles

**Affiliations:** a Faculty of Industrial Design Engineering, Delft University of Technology, Delft, The Netherlands; b Department of Orthopaedic Surgery, Reinier de Graaf Hospital, Delft, The Netherlands; c Zimmer Biomet Europe BV, Dordrecht, The Netherlands

**Keywords:** Team interactions, hospital wards, learning history, healthcare design, design thinking

## Abstract

**BACKGROUND:**

Supporting teamwork in healthcare is a way to foster both the quality and safety of care, and better working conditions for all the team members. Although increasing attention is paid to this topic on a general level, there is less knowledge about its unfolding in orthopaedic units and its translation to interventions.

**OBJECTIVE:**

To identify concrete opportunities for teamwork intervention through a design thinking approach by analysing the teamwork dynamics of an orthopaedic team.

**METHODS:**

An adaptation of the learning history method, comprising shadowing, observations and interviews involving 26 orthopaedic team members at a top clinical teaching hospital in the Netherlands, was applied. A thematic analysis was conducted to derive themes that describe team dynamics and to subsequently extrapolate opportunities for intervention.

**RESULTS:**

We identified five themes and translated them into four design opportunities for intervention, namely: a) Improve daily rounds by reducing cognitive overload and promoting confidence; b) Improve collaboration by building empathy; c) Connect the patient with the professional team; and d) Support changes by fostering learning. Suggestions for concrete actions are presented for each opportunity.

**CONCLUSIONS:**

Opportunities to improve teamwork among healthcare professionals, specifically those in orthopaedics, revolve around the creation of common knowledge, the fostering of mutual understanding, and the design of tools and activities that support these processes.

## 1 Introduction

In recent years, increasing attention has been paid to teamwork dynamics in the healthcare domain [1] because communication and teamwork failures contribute enormously, if not primarily, to the causes of adverse events (AEs) that result in involuntary patient harm [2]. A well-coordinated team produces several benefits, for instance fewer inefficiencies and a lower staff resignation rate, thus better working conditions for all the stakeholders [3]. Moreover, patients who experience a good team coordination are more satisfied with the care they receive [3]. Therefore, enabling and supporting teamwork is a way to foster both safety and the quality of care. This paper focuses on teamwork dynamics in healthcare, taking as case study an orthopaedic team, and identifies design opportunities for interventions aimed at improving teamwork.

### 1.1 Teamwork in the healthcare context

Teamwork is defined as an “Adaptive, dynamic and episodic process that encompasses the thoughts, feelings and behaviour among team members while they interact toward a common goal” [4]. This means that besides task performance, teamwork is a necessary component of teams, since it represents *how* the tasks are performed by the team members interacting with each other. In particular, Salas and colleagues recognise six core processes — coaching, conflict, communication, cooperation, cognition, coordination — and three influential conditions — culture, composition and context — that describe processes of teamwork. These are all factors that must be holistically taken into consideration to ensure successful team performance [4].

To relate these factors to the objective of this study, it is necessary to introduce the characteristics of the healthcare context. This setting is a complex system composed of a variety of interdependent components, such as the existence of several procedural routines or of parallel patient care goals [5]. At the same time, it is a constantly changing environment that is under time pressure and is not fully predictable even when applying standards. For example, the way patients respond to cures may differ in terms of recovery time, type of needed medicines or pre-existing patient conditions which require to tailor protocols. These factors make the system challenging to work in at both an individual and a team level [6]. Moreover, the context is specifically characterised by multidisciplinary and fluid teams, meaning a changing composition of the team due to, for example, shifts [6, 7], and relies on a multi-team system, composed of a variety of sub-teams interacting interdependently [5, 8]. Multiple decision-makers are present which implies a need for alignment on actions to take, this way ensuring that the singular professional perspectives towards a care plan work in synergy to the ultimate patient care goal. In healthcare, the various professions historically worked in clearly delineated sectors, each with its specific skills, expertise and training [2, 5]. This led to hierarchical organisations [5, 7] with specific implications and problematics in relation to teamwork dynamics. A strict hierarchical differentiation can impair open communication, especially when it comes to raising concerns and discussing differing opinions constructively [1–3, 5]. In light of the team members’ different backgrounds, it is necessary to create a shared understanding of each other’s roles and objectives as well as facilitating awareness of the situation [4]. Situation awareness (SA) refers to how a person perceives, comprehends and projects data from the environment he is presented with. Therefore, it represents the basis on which decisions are taken [9]. When individuals work together with inter-related tasks and different backgrounds, like in healthcare, each individual’s SA needs to align and form a shared SA. This way, teams are provided with a common ground to coherently act upon [9].

Factors such as stress or workload, individual’s goals and expectations, as well as experience and skills, influence the accuracy of the situational awareness each team member can create [10]. Moreover, the means the caregivers use to communicate with each other influence their relationships and the resultant quality of their communication. For example, the increasing use of electronic records and other media such as emails facilitates faster, non-stop communication but also reduces face-to-face interaction [1, 3]. This lack of gestures, intonation and facial expressions can lead to misunderstandings [3] and the greater dispersion of team members [1]. In complex environments, users can also face data and technology overload, for which maintaining SA is even more important and more challenging [9]. In fact, developing a thorough understanding of the situation is necessary to comprehend the complexity of the system and the consequences of actions, but it is a very demanding task due to the great amount of factors to take into consideration [9]. A shared understanding within the team in a healthcare setting is paramount, but achieving such an understanding is a complex process.

### 1.2 Teamwork in orthopaedics

Research in the field of orthopaedics suggests that supporting and fostering teamwork is a way to achieve safer care, which is in line with the research on the general healthcare setting. Two studies in particular [11, 12] investigated the causes of adverse events (AEs) in orthopaedic departments and identified teamwork as one of the main culprits. According to the first study [11], the majority of the AEs involved the entire staff around the patient failing to follow protocols, policy or guidelines. The authors suggest fostering multidisciplinary work by establishing standard procedures, organizing team training sessions, understanding different professional cultures and ensuring proper communication. In the second study [12], 43% of the AEs recorded over a four-year period (2005–09) were due to poor situational awareness, poor decision making, poor leadership or poor communication. In both cases, the majority of AEs — including those with medical causes (e.g. infections) and those resulting from a lack of non-technical skills, such as coordination or communication — occurred in the peri- or post-operative phase. These studies analysed the causes of AEs and discovered that teamwork was one of the factors leading to accidents. Although they did not explicitly cover reasons for poor teamwork in the first place, they do underline the importance of teamwork in orthopaedics, highlighting the need to tackle the topic in the whole care process. Despite the results of these studies, there is still little research on the overall processes of orthopaedics in relation to teamwork dynamics, and thus on what specific contextual factors hinder or foster teamwork [1, 3].

### 1.3 A design thinking perspective

Teamwork implies the consideration of behavioural aspects of the interaction among team members while performing their tasks. Thus, identifying teamwork dynamics requires a holistic approach that can unravel human and contextual factors contributing to poor or good teamwork. A discipline whose methods address questions from such a perspective is that of design thinking [13]. The method comprises five activities: empathise, define, ideate, prototype and test [14]. A human-centred perspective is applied to explore people’s motivations behind what they do or do not do. This means empathising with the users: experiencing and feeling situations the way they do. Inspired by these first-hand experiences, the problem to be solved can be defined and solutions can be generated to address opportunities in an innovative way. Prototypes are tested through simple means, such as paper mock-ups, to gather information about which ideas work better and how the users would use the new designs [13]. The five steps do not necessarily constitute a linear approach, but can be applied in an iterative fashion, for example by using prototypes throughout the project to explore the context [14].

One representative example of a successful application of this process in healthcare is that of Kaiser Permanente’s hospitals, based in the United States, which established an internal Innovation Consultancy in collaboration with the design firm IDEO [15]. In one project, they observed the exchange of information among nurses between shifts, and realised it was unstructured, unreliable and time consuming, reflecting a ‘hole’ in the care process as felt by the patients. These sorts of insights helped them to uncover the real problems and to address them with a series of solutions, such as involving patients in the discussion or using a standard digital format. Another example is their medication error project, where observations led to unfold the problem of distractions. Co-creation and prototype testing with users generated and evaluated ideas to solve the issue. For the sake of our study, we followed the design thinking approach in order to suggest concrete solutions for intervention.

### 1.4 Research aim

The aim of the present research was to analyse the teamwork dynamics of an orthopaedic team, looking at behavioural and environmental elements, in order to identify opportunities for teamwork interventions. To do so, we adopted a design thinking approach and looked closely at the team members’ experiences to promote the ideation of solutions. In this paper, we present the results of the first two steps (i.e. empathise and define) of a broader project that was aimed at improving teamwork in the orthopaedic team by design and where all five steps were followed. For further results, we refer to Caprari, 2016 [16]. This paper continues with the description of a qualitative study that was carried out with an orthopaedic team at a Dutch hospital. The results section provides concrete insights for teamwork interventions in this context through the definition of themes and consequent opportunity areas. The paper ends with a proposal for further research and a reflection on its contribution and limitations.

## 2 Method

### 2.1 Learning history method

To enable us to experience the stakeholders’ perspective first hand, we chose to base our study on an adaptation of the learning history method [17], which is a research and analysis process originally developed for organisations to outline learning opportunities based on past events [18]. In this process, members of the organisation collaborate with the researchers to identify ‘noticeable results’ (e.g. a monetary loss) that are related to opportunities for improvement. The researchers then conduct interviews with the various stakeholders involved in each event, thus gathering a variety of perspectives. In these open conversations participants reflect on their experiences, often making new connections through which tacit knowledge surfaces [17]. The method finally entails the collection of the various perspectives in a document called a ‘Jointly Told Tale’, which, for each event, combines both the stakeholders’ perspectives and the researcher’s interpretation. In line with the research aim, this method provides the space to understand the organisation dynamics and gain in-depth knowledge of its members’ experiences.

We used this methodology as the basis for both data collection and analysis, adapting it to our context. First, tangible outcomes were identified by the researchers by shadowing orthopaedics professionals; thus, the starting points for discussions were the researchers’ observations, rather than pre-identified events. This is because teamwork dynamics are not exactly measurable (unlike a monetary loss). It was therefore important to let the researchers experience the team members’ behaviour in relation to teamwork in order to identify tangible results. Repeating observations and interviews with 26 participants provided evidence of patterns in teamwork dynamics, and hence the possibility to discuss the same topics from various points of views. A thematic analysis was then used to create the jointly told tale of this research. In section 2.5 we provide a detailed explanation of the method.

### 2.2 Setting and standards

The study was carried out in the orthopaedic department of a top clinical teaching hospital in Delft, the Netherlands. Most of its operations are elective surgeries, such as total hip or knee arthroplasty (THA, TKA). The orthopaedic team is composed of seven orthopaedists and seven residents, rotating from outpatient clinic to operating room (OR), and one or two medical students. Two specialised nurses and one physician assistant (PA) are responsible for patients’ education and follow-up visits. A research nurse collaborates with doctors and PAs in conducting trials. On the ward, a team of around 20 nurses work closely with two orthopaedic physiotherapists. The nursing team varies in composition and size, since the teams are deployed in three shifts (each with three or four nurses, except for the night shift, during which only one nurse is present) and need to meet the demand for care. In addition, given the decreasing length of stay (LoS) of the orthopaedic patients, the ward had recently undergone a reduction in the number of beds (to 12) and a merger with another ward specialising in urology, ear, nose and throat (ENT), dental surgery and gynaecology, grouped under the name of ‘minor surgeries’ (in Dutch, *kleinsnijden*). In this context, an orthopaedic patient’s typical journey involves several stakeholders. In the case of a total hip replacement, during the period between diagnosis and surgery (approximately two to six months) the patient generally meets the orthopaedist (diagnosis), the specialised nurse (patient education), the anaesthetist (pre-operative screening) and the ward resident (pre-operative final check). When the patient is admitted for the surgery, nurses and physiotherapist visit the patients individually or together with the ward doctor. In the follow-up phase after discharge, patients continue rehabilitation on their own and also meet the hospital caregivers again, that is, they meet the PA or specialised nurse two weeks after the operation, the surgeon after six weeks, the PA again after three months and the surgeon again after one year.

### 2.3 Participants

The study had a total of 26 participants (see Table 1); one of them (a ward doctor) participated in three activities, and three others (a nurse, a physiotherapist and another ward doctor) took part in two activities. In order to investigate the overall team dynamics, 10 stakeholders were chosen such that at least one of them represented an orthopaedic profession or a stakeholder group. Nurses, who comprised the biggest sub-team, had two representatives with different experience levels. As Table 1 shows, a patient and an anaesthetist were also included: the first to experience the team from the patient perspective, the second as playing a key role in the process but not exclusively connected to the orthopaedic care unit.

**Table 1:** Overview of participants and research activities

Activity	Participants	Gender	Location
Shadowing + interview	Ward doctor (resident)	M	Ward, Out-Patient clinic
	Patient	M	Operation room, Ward
	Nurse (less experienced)	M	Ward
	Physiotherapist	M	Ward
	Physician assistant	M	Out-patient clinic
	Nurse (more experienced)	F	Ward
	Anaesthetist	M	Operation room, Ward
	Specialized nurse	F	Out-patient clinic
	Research nurse	F	Out-patient clinic
	Surgeon	M	Operation room
Interviews	Ward doctor (resident)	F	−
	Nurse	F	−
	Ward manager	F	−
Observations	(*Daily rounds*)		Ward
	3 nurses	3F	
	1 ward doctor (resident) (*Multidisciplinary round*)	F	
	1 physiotherapist, 5 residents (1 as ward doctor), 1 surgeon, 1 psychologist, 2 nurses	5M, 4F	Ward

All participants voluntarily participated in the study. The researchers complied with the hospital research and employee code of conduct, as they themselves are registered hospital co-workers. In the case of the professionals, the researchers verbally informed them about the study and anonymised all data used for analysis and communication purposes. Before shadowing the patient, he signed the hospital code for the collection and treatment of data. During the observations, the researcher wore a white coat, like that of the nurses, in line with the hospital’s regulations.

### 2.4 Data collection and analysis — adaptation of the learning history method

**Fig. 1:**
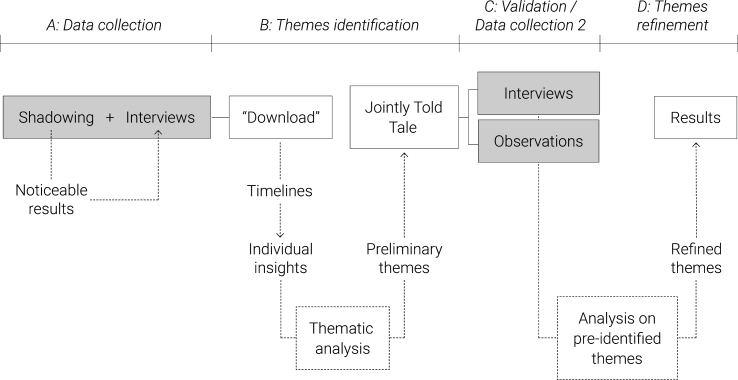
Schematic representation of the research and analysis process followed in the study. The process is based on the learning history method and was adapted to the research need.

The study followed the process outlined in Fig. 1. The overall process was divided into two phases of data collection, both of which contributed to the final results.

**A. Data collection:** The participants in the first part of the data collection were shadowed for a complete day/shift. Data were collected in the form of notes, using a timeline that structured the observation according to teamwork activities, barriers and enablers, as well as tools used for specific tasks. Throughout the day/shift, events that suggested potential points of intervention for teamwork dynamics (i.e. the noticeable results) were selected for discussion with the participants in a semi-structured interview at the end of the shift (or the following day). The interviews consisted of open-ended questions aimed at investigating observed barriers and enablers. For example: ‘What do you think of the day evaluation activity?’ or ‘The multidisciplinary round seemed quite fast paced. Do you encounter any difficulties? What type?’

Additionally, during the interview, a map made of concentric circles was used to support a conversation concerning the users’ team perception and relationships. Participants were asked to map the closest and least close team members in their daily practice and explain the reason for these choices. Interviews were audio-recorded for analysis.

Spending a full day with the participants enabled the researcher to empathise with them and understand their tasks and goals, as well as their feelings and motivations. This is a necessary step to increase knowledge of the user’s world, namely by deeply immersing oneself in their experience without judgement [13, 19]. Moreover, the participants’ specific type of work led to an exploration of various working environments, such as operating room, ward and out-patient clinic, offering the chance to also observe the environmental factors that influenced the teamwork.

**Fig. 2:**
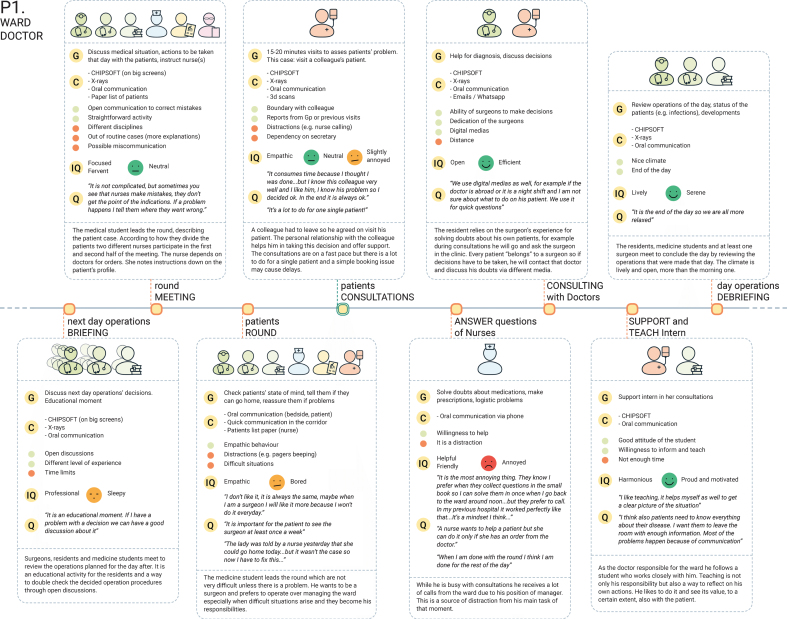
Example of a timeline where the data were organised after their collection on paper notes.

**Fig. 3:**
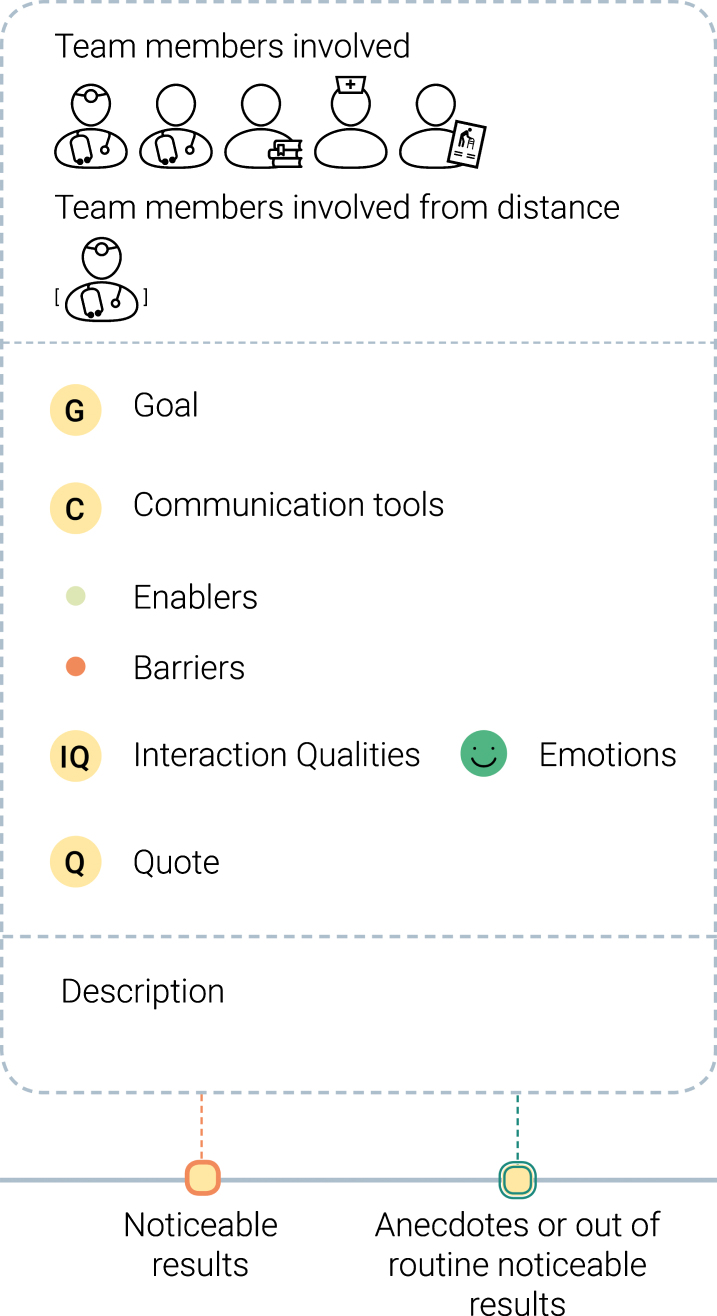
Explanation of how the data were structured for each event reported in the timeline.

**B. Themes identification:** The observation notes and the audio-recordings were used for the “download” of the insights in “user boards” [20]. We collected quotations and observations and structured them according to emerging categories in relation to the research aim, such as team perception, team relationship, and barriers and enablers. This activity was carried out at the end of each interview, in order to quickly identify topics that could be sought or asked for in other interviews or shadowing activities. To serve as an organised dataset, 10 timelines were created representing noticeable results on teamwork dynamics that each specific participant experienced over a day or had experienced in the past. An example is provided in Fig. 2. Details about how the data were structured are presented in Fig. 3. The timelines organise the data according to the team members involved, the communication tools used, the barriers and enablers, as well as interaction qualities and emotions of the narrator, quotations and a summary of the event.

Next, a thematic analysis of the individual insights was carried out to identify preliminary themes. The themes were chosen by the researchers as “those factors considered relevant to answer the research questions” [21], thus recurrent issues and/or enablers that influenced teamwork dynamics. The impact of specific dynamics was also taken into consideration; therefore, a theme could also include a non-recurrent but impactful issue, chosen on the basis of the obtained knowledge of the team chain of actions. The ‘Jointly Told Tale’ of the orthopaedic team could then be compiled, where each theme was explained, supported by different team members’ quotes.

**C. Validation/Additional data collection:** When the preliminary themes had been formed, we reviewed them further on the basis of their impact on the team. Since two of them were based on critical but non-recurrent facts (i.e. hierarchical pressure, misalignments within the new team), it was decided to validate these aspects with supplementary research, in order to obtain a deeper knowledge of the issue and an ampler set of opinions by interviewing or observing additional participants. Thus, three interviews and two observations were carried out with the stakeholders involved in those themes, using guidelines created on the basis of the pre-identified issues. Interviews included team interaction questions (e.g. what were input and output of daily rounds and how they were communicated), and behavioural ones (e.g. recalling and narrating an episode of hierarchical pressure and define what behaviours of the counterpart made the situation uncomfortable, what emotions it elicited and how the situation was solved). Interviews were audio-recorded and observation data were collected in the form of structured notes.

**D. Themes refinement:** Interview recordings and observation notes from the extra data collection were analysed according to the themes previously identified, by adding evidence or nuances within a theme. These refined themes were eventually narrowed down as a result of this research and used to identify opportunities for teamwork intervention.

## 3 Results

**Fig. 4:**
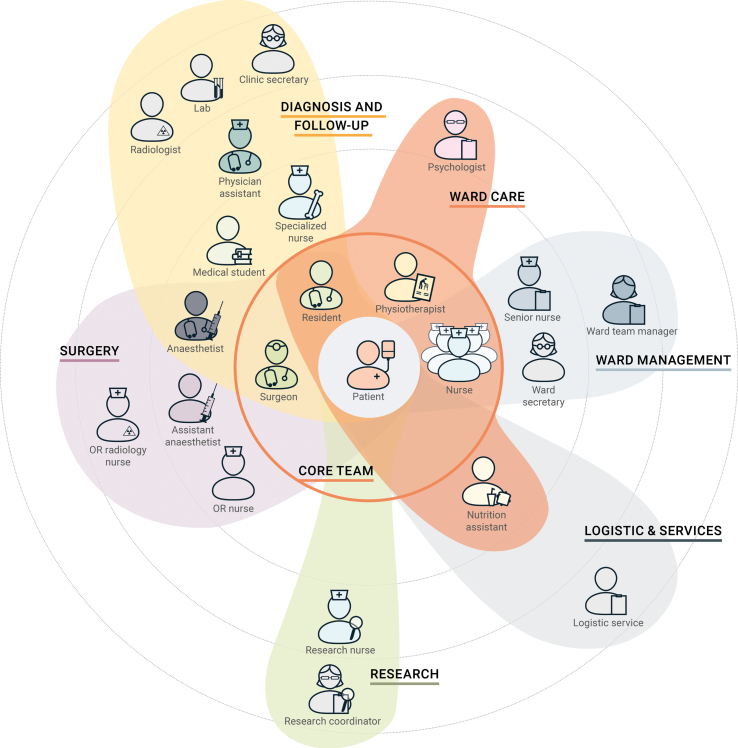
Representation of the team members in a map showing sub-teams based on the stages of the care process.

The shadowing and the interviews allowed us to acquaint ourselves with the team composition and structure, which helped us to understand the dynamics among the team members. To identify the team structure, we did not look at the classic role-based division, but at how the team members actually formed groups according to the stage of the care process. This enabled us to create a realistic visualisation of relationships and connections, whereby a team member can belong to multiple groups depending on what stage the patient is in. As shown in Fig. 4, we categorised the team into seven groups: diagnosis and follow-up; ward care; ward management; logistic and services; research; surgery; and core team. In this representation, team members are more or less close to the patient (who is positioned at the centre) according to how much time they spend with him throughout the process. This approach outlined which professionals can be placed in closer contact with the patient, constituting an inter-professional core team.

### 3.1 Themes

We identified five themes that describe relevant teamwork dynamics of the orthopaedic team: (1) Influence of attitude and environment on communication between nurses and residents; (2) Need for mutual understanding; (3) Different aims of briefing activities; (4) Patients’ role within the team; and (5) Impact of organisational changes on team dynamics.

**Theme 1: Influence of attitude and environment on communication between nurses and residents**

The collaboration between nurses and residents was found to be of primary importance for the ward care. Nurses monitor the patients, collect and provide information to the resident, who issues instructions for the patients’ care plans. In our particular study hospital, each resident is in charge of the ward for one month, on rotation. Both our observations and the professionals’ quotations revealed that there are attitudinal factors and misunderstandings as well as environmental, task-related elements that hinder smooth collaboration.

1a. Attitude factors and misunderstandings

In the category of attitudinal misalignment, we included examples of behaviours and mind-sets that were identified as negatively influencing the interaction between nurses and residents. An example is nurses encountering instances where they do not feel that their opinion is valued by the residents: they prefer it when doctors make them feel comfortable and show an open attitude by listening to their judgment. In some instances, the situation can be a case of explicit negative exercise of hierarchy. An example was provided by the ward manager regarding an intervention to mitigate a conflict.

“Surgeons value what we do, what we say, what we think. Young doctors sometimes don’t listen. Some are easier to connect with than others. It’s all about their behaviour, whether they make me feel comfortable.” — Nurse

“In urology, sometimes I had to step in because of a doctor shouting at the nurses!” — Ward manager

Besides behavioural aspects, various nurses and the ward manager reported that having well-defined protocols to follow allows them to feel confident about their judgements, and thus support patients, especially when such conflictual situations arise. On the other hand, doctors become frustrated when nurses appeared to not fully comprehend instructions and their objectives. These misunderstandings resulted in frequent phone calls for clarifications that caused distractions and overload for the doctor, but could also lead to mistakes (e.g. communication of wrong information to the patient).

“Sometimes the nurses don’t understand us, they don’t get the point.” - Resident

The way information is communicated influences the occurrence of misunderstandings. For example, residents reported that experienced nurses are able to convey information to the point, in a more structured manner, compared to less experienced nurses. They appreciate this skill, especially when they are on a tight schedule, since the information comes across in a way that helps them take decisions faster, by receiving organised input.

“Experienced nurses are much more to the point; they know what we want to know.” - Resident

*1b. Environmental* and *task related factors*

Our observations revealed factors that do not directly depend on individual personalities and skills, but depend on the environment and its organization, such as furniture arrangement, and the tasks that need to be accomplished. For instance, we observed these kinds of factors at the key moment when the exchange of information happens: the daily rounds. Nurses prepare for this meeting by reading the patients’ files (usually four each) that nurses on previous shifts filled in. This is a textual report structured by the ABCDE (airway, breathing, circulation, disability and exposure) method [22]. After the meeting, the doctor and the nurse visit the patients to communicate the decisions they have taken. During the meeting itself, we observed that nurses and doctors often sat with their backs to each other. This was most likely due to a combination of the arrangement of the furniture in the room and the tasks to be carried out. In fact, both of them have profession-specific screens to look at and interact with, thus the need for two computers that were not always positioned next to each other.

Moreover, nurses divide their attention between notes on paper and the digital report to retrieve information, while doctors listen to them and look for x-rays or examination results. Both doctors and nurses need to write down what has been said as a summary (one for doctors and one for nurses) and later log it in the system. Due to this multitasking, the conversations were often fragmented and cognitively overloaded. This scenario became even more evident in the multidisciplinary round, which was held once a week and lasted for half an hour, as it had to cover all 12 patients. Since this was a bigger group (10 professionals) with a shorter time frame, the nurses had to communicate even more effectively and efficiently, whereas their tasks remained the same, putting them under pressure.

“In the big round I feel a bit the hierarchy. It’s a big group and it all goes so fast… I barely have time to give my input.” — Nurse

Another effect of time restrictions and established routines is that nurses feel that doctors tend to overlook how a patient feels. Of all the stakeholders, nurses spend the longest time in close contact with patients and tend to have a more holistic view of their wellbeing. Discussing patients’ feelings, considering this topic as part of the overall picture is relevant for nurses. However, this information is often overshadowed by time limitations and the relatively less importance of this data within the clinical discussions, except when there is a specific problem arising from a patient’s psychological state.

“They always ask about the pain but not how the patient feels.” — Nurse

“We simply don’t have the time to really talk with all patients.” — Resident

**Theme 2: Need for mutual understanding**

During the interviews and the discussions about the relationship maps, we noted several instances that show how a smooth collaboration is related to understanding each other’s needs and motivations, as well as to creating opportunity for support.

2*a. Sharing knowledge*

A recurrent factor was that a fundamental element of mutual understanding is sharing the same knowledge. This is easier for those who share the same profession, as their backgrounds are similar, but even across disciplines the collaboration is smoother when team members are aware of what information the others need, what their tasks are and what goals they have to achieve.

“I can say anything to them [the other anaesthetists] because they understand me and they have the same problems I have.” — Anaesthetist

“Physiotherapists understand us. They know what I need and I know what they need.” - Nurse

Creating such understanding within multidisciplinary teams was found to be challenging. Profession-specific cultures may hinder reaching a common understanding, especially if the communication of objectives is not clear. A concrete example in our case was the introduction of the Rapid Recovery programme, a new orthopaedic care approach that led to a reduced length of stay (LoS). At the start of the programme, the accelerated care path was difficult to accept from a professional perspective, especially for the nurses, who were sceptical regarding a short stay. The same happened when the LoS was eventually reduced to one day, but with the passing of time and good results, the new methodology became clear and simply one of the various procedures.

“In the beginning we had nurses saying ‘Why are you making him walk? He’s just been operated on!’ — with the patient there [in the same room].” — Physiotherapist

The process of creating mutual understanding not only takes time, but also requires the development of personal bonds and experiencing the other’s working attitude. Such a process influences team interactions, in particular when it comes to trusting one another. This was especially observed in teams that collaborate closely, such as teams of nurses, who rely on mutual support and often delegate tasks. Hence, although sharing the same profession helps, it does not automatically result in effortless cooperation.

“I need time to find out how she works. Is she reliable? Does she have a lot of experience? For me that is very important.” — Nurse

2b. Establish the right climate to reach out for support

The majority of the participants mentioned that they experienced a low hierarchical differentiation. The main advantage was being able to easily approach colleagues (or supervisors) for support or consultation. This was found to be a key element both in the outpatient clinic (e.g. residents and physician assistants approaching a surgeon) and on the ward (e.g. nurses being encouraged to propose care plans to the resident, even though, as seen in the previous theme, it was not always a flawless process). According to participants, the open attitude makes the team collaboration also visible to the patients, contributing to making them feel taken care of.

“There is no problem to go to the doctor, it’s not a problem to discuss with him and he doesn’t make it a problem to go to the patient together. The patient finds out that we are a team. It looks professional. This is our opinion, as a group, you are not just a number to us.” — Physician assistant

**Theme 3: Different aims of briefing activities**

Throughout our observations, we noted how the practice of briefing and debriefing was spread amongst the sub-teams. Although these activities have the common purpose of aligning the team towards the tasks they will perform, differences are present that, in our opinion, are valuable in order to determine opportunities for improvement.

The nurses have a resource-oriented start to their mornings. On a board, they express their expectations for the day, discuss concerns or arrange logistic matters. On the basis of the discussion, a green, yellow or red smiley is placed on the board. In the case that any personal problem might hinder their performance, it is reported too. At the end of the shift, the team gathers to evaluate the day, reporting points for improvements and positive practices they encountered throughout the day, usually logistic oriented.

Residents, surgeons and medical students have a morning briefing regarding the following day’s surgeries, with a focus on clinical procedures. This briefing is also an educational moment for residents and students, and thus open discussions are encouraged. The conversation is supported by X-rays and the EHR system visualised on a large monitor. Even in their case, a debriefing activity happens at the end of the day to review the outcomes of that day’s surgeries.

“The morning brief is an educational moment. If I have a problem with a decision, we can have a good discussion about it.” — Resident

In the OR, the whole team participating in the operation conducts a safety-oriented activity, based on a checklist for exchanging information regarding the patient and the procedure. This briefing happens before and after the operation (called time-out and sign-out, respectively [23]). At the end of the day, the team also performs a day evaluation, whereby they grade their day with a score ranging from ‘excellent’ to ‘poor’ and make suggestions for improvement. However, because this method does not specify what exactly is to be graded, the surgeon evaluated the process as ineffective.

“It’s not the most effective way because we don’t know what we’re grading. You had a good day, but what does that mean? What should we score?” — Surgeon

**Theme 4: Patients’ role within the team**

During our observations, we noted that the patients had different ways of engaging with their care and consequently with their caregivers. This led us to investigate the professionals’ view on the patient’s role in the team. Most of the professionals considered the patient the subject or goal of the team, rather than an actual team member. However, they did recognise that the patients are the reason the team exists in the first place and that they are the ones who set the standard, by wanting optimal care. To a certain extent, this consideration identifies the patient as ‘the leader’ of the team.

“He’s not part of the professional team, but he has an important role: without him there is no team.” — Anaesthetist

“I think the patient is the subject of the team and he is the leader of the post-operative part.” — Physician assistant

For some professionals, this is more evident because they clearly see the patient’s role in defining their interaction with them. For instance, physiotherapists or nurses can experience patients’ attitudes and different levels of dedication when carrying out rehabilitation exercises, which influences the type of work they need to perform with the patients. Therefore, team members adapt to the type of patient, for example with regard to the amount of information they give and the strategy they use to communicate with the patient. Nurses or physiotherapists, who have time to create stronger bonds with patients, develop a strategy of communication, for example, to stimulate someone who is not exercising enough.

“This is not a hotel! They have their part to do too: rehabilitation. Some stay a long time and they try but it doesn’t work, while others have the ability but make less effort, so I try to explain to them why it is necessary for them to move.” — Nurse

“I accept that not everybody reacts in the same way. Sometime you lose, sometime you win.” — Physiotherapist

Other specialists may tend to be less involved and accept the patients’ will, even if it is not exactly aligned with the recommended protocols. Some other patients are really engaged with their care, do research on the Internet or often come to appointments with notes. The professionals appreciate this approach, but are also concerned about the amount of time that is sometimes required to discuss certain questions, which may be the result of misleading information the patient came across while doing research.

“The patients chose to have this operation. If they don’t read the information material, it isn’t ‘my problem’.” — Specialised nurse

“They’re curious and want to know everything. I like that — as long as it doesn’t take 30 minutes to answer irrelevant questions (like if they found some crazy thing …). — Physician assistant

Although the professionals’ approaches may differ, aligning the communication strategy was found to be contributing to patients’ satisfaction. In fact, various participants stressed how communicating consistent information throughout the process is necessary to manage patients’ expectations and create the right mind-set for recovery. Considering patients beyond their medical problem is also a way to make them feel comfortable, thus increasing their wellbeing for recovery. The whole team should therefore be aligned towards this goal.

“One important factor is considering the patient as a person, not just a hip that does or doesn’t function. Provide him with consistent information upfront so that he feels confident. Everybody should be aligned towards this goal.” — Physiotherapist

**Theme 5. The impact of organisational changes on team dynamics**

All the participants had been affected in some way by the organisational changes introduced a short time before our research. The rearrangement of the hospital according to a flexible concept resulted in the unification of orthopaedics and minor surgeries on one ward with a reduced number of beds, as well as in a new use of spaces and resources, such as the elimination of the residents’ room and of fixed work stations.

5a. Effect on the nursing team

These changes influenced the team interactions, and especially those in the nursing team. First, many experienced nurses left and new colleagues were introduced, as were flexible nurses who would rotate across departments. Therefore, the team composition changed. This illustrates how this factor has a major influence on cooperation and team performance. Those who stayed had to adapt to new working routines and rebuild the team with their new colleagues, thus having to make an effort to change after years of well-functioning habits. Besides changes in composition, also a change in roles was required since colleagues from the other specialities had to be included in the orthopaedics work, and vice versa. Nurses had to learn new skills, and teach others their skills. For the most experienced ones, such a request can constitute a radical change that, especially at the beginning, resulted in stress, work overload and an understaffed team.

“I chose orthopaedics. I like the kind of patients and work, so they can’t expect me to do something I don’t like.” — Experienced nurse

“We have lots of new colleagues now… I feel like I’m responsible for the whole thing.” — Experienced nurse

Since the nurses are basically the main presence on the ward, problems within this sub-team affect other team members. In a few instances, the physiotherapist encountered mistakes made by the new nurses, such as giving incorrect information to patients or making errors in the procedures. Compared to the previous status, it represented a decrease in performance.

“It’s a pity that very experienced nurses have gone and you see the new ones make procedural mistakes. Before it was like a train — everybody knew what to do. I think it will take time before it is good again.” — Physiotherapist

5b. Effect of the spatial arrangements

The second element of the organisational change — the use of flexible spaces — also offers points for reflection on team dynamics. For example, residents identify themselves as a united group, since they are all learning and rely on each other for support. Having a room for themselves provided a means for nurturing their team bonding, beyond their working relationship, which was now missing.

“We used to have a room and it was good. It’s not only the room, it’s the time you spend with your colleagues.” — Resident

## 4 Design implications: Opportunities for teamwork interventions

Having acquired a deep understanding of teamwork dynamics of the orthopaedic team and of the experiences of its members, we derived from the results four opportunities for teamwork intervention: a) Improve daily rounds by reducing cognitive overload and promoting confidence; b) Improve collaboration by building empathy; c) Connect the patient with the professional team; d) Support changes by fostering learning.

### 4.1 Improve daily rounds by reducing cognitive overload and promoting confidence

Considering *theme 1*, a point of intervention is to improve the exchange of information between nurses and residents during the daily rounds, on both a task and a behavioural level. Our results show that their collaboration is hindered by attitudinal and environmental factors, encompassing the use of hierarchy, misunderstanding of goals, time limitations and multitasking. In terms of behavioural and emotional aspects, this opportunity for intervention concerns: 1) addressing the nurses’ need to feel confident about procedures in order to have the tools to advocate for the patient when needed; 2) explicitly clarifying goals and objectives as a way to reduce miscommunication and additional workload; 3) using guidance for the communication of information in such a way that nurses have support in recalling all the relevant information whilst providing structured data to the doctors for decision making. This last point overlaps with time limitations as a task-related factor that hindered the communication of, for example, patients’ feelings. Creating a better structure could allow time to be managed more effectively. On an environment level, there is an opportunity to change behaviour by changing how the room is organised (e.g. arrangement of tables).

#### 4.1.1 Suggestions for intervention

Concrete suggestions for intervention include the design of tools for exchanging information, in particular between team members from different professional backgrounds. For example, the EHR system could have a unique screen for the daily rounds, shared by nurses and doctors. Such a page could collect an overview of the information that is usually covered during the meeting (e.g. vital signs, wound leakage, etc.) and could structure them with visual cues rather than textual information. This would make data easier and faster to read for the doctors, whilst supporting the nurses in presenting the patient’s situation in a structured manner. For professionals with less experience, this kind of tool could provide guidance and support, making them feel confident about their assessment based on the overview they are provided with. Having all the relevant data in one place reduces the team members’ need to check information somewhere else (e.g. in previous reports), thus reducing multitasking and, consequently, cognitive load. This guidance should be supported by the layout of the room, for example by allowing people to sit facing each other.

### 4.2 Improve collaboration by building empathy

A second opportunity for intervention is to empower team members to increase their understanding of each other’s needs, roles and goals. *Theme 2* showed that professionals had a positive experience of collaboration when the other members understood their needs and acted consequently, showing empathy. This was the case in multidisciplinary teams as well as within professions. Building personal relationships and experiencing how a colleague works were also identified as factors that contribute to feeling confident about trusting colleagues. Hence, this opportunity covers strategies for team building based on fostering shared knowledge and empathy for each other’s roles with the aim of improving collaboration.

#### 4.2.1 Suggestions for intervention

An example of an intervention could be to use shadowing activities to let team members put themselves in their colleagues’ shoes. For example, residents could spend a day following nurses and being given specific tasks that are aimed at making the residents empathise with the nurses’ experience. Shadowing guidelines could be designed ad hoc to stimulate particular observations (e.g. moments when a nurse needs information for which she would call a doctor), thus challenging the residents with thought-provoking tasks. Such activities could be extended to the whole team, by first analysing frictions between given team members and then preparing shadowing guidelines. At a later stage, it could also become a way to identify teamwork flaws throughout the process by the team members themselves, creating awareness of the topic and opportunities for improvement. One key point would also be to provide guidance on reflecting about teamwork factors, since, as seen from theme 3, to be considered effective reflections should be guided towards a specific goal. Asking team members to notice how colleagues are able to convey clear information, or what behaviour they show when interacting with other team members, could be a way to do so.

### 4.3 Connect the patient with the professional team

A third opportunity for intervention concerns the role of the patients within the team. In *theme 4* we showed how patients are seen as the subjects of teams, rather than as team members. However, they are the leaders of the team in the way they dictate the standards and define the type of work and communication strategy team members need to apply. This means that they influence the teamwork depending on the level of engagement in the tasks they are required to perform. However, different professionals do not always adapt to different patients’ profiles in the same way. For this reason, there is an opportunity to align team members’ interaction with specific patients’ profiles, in order to deliver patient-centred care.

#### 4.3.1 Suggestions for intervention

To use this opportunity, the patient can be considered the connecting point among professionals to ensure the alignment of care. Tools can be designed to accommodate the patient’s profile and the communication strategy a caregiver can use. For instance, for those patients who seek to be in control and are willing to play an active role in the team, tools can be designed that enable them to organise their plans and the information they receive. In parallel, caregivers can have a dedicated tool for communicating information based on the same principle. Aids should take into account that the communication is always between caregivers and patients; thus, both sides should be enabled to effectively collect and share meaningful information for the counterpart. This means providing patients with clear and layman explanations of treatments, recovery times and so on. These tools can play a role for preparing a consultation but also be used during the consultation itself to facilitate and support the communication between patient and care-giver, for example with the use of graphical visualizations.

### 4.4 Support changes by fostering learning

A fourth intervention concerns the need to support caregivers in adapting to changes. In *themes 2 and 5*, we reported specific cases of difficulties in implementing changes, specifically the adoption of the Rapid Recovery programme and the merging of the two wards. In the first case, the introduction of new procedures was not easily accepted, until results were delivered and clearer methodologies had been developed. In the second case, however, we outlined how an imposed change in team composition led to dissatisfaction, stress and the need to rebuild a well-coordinated group. At the same time, the nursing team were asked to learn new skills and teach theirs to new members of the team. This opportunity encompasses various aspects of adapting to changes: making the goals of new procedures explicit, discussing team members’ expectations regarding changes, acknowledging the team learning need and creating an atmosphere for sharing learning throughout the process.

#### 4.4.1 Suggestions for intervention

Applied examples could be to use boards for briefings and debriefings with an orientation towards documenting cases for learning. Especially for nurses, who work closely and rely on mutual support, creating an effective learning environment helps new nurses increase their task and procedural knowledge, which is also a way to create professional bonds and better teamwork. As seen in theme 3, in certain sub-teams such as that of residents, the learning need is acknowledged, hence their activities, starting from the briefings, are oriented towards increasing their knowledge. If the nursing team is in the process of changing, we suggest acknowledging the team learning need and creating moments to share what has been learned. One instance can indeed be to utilise the briefing moments. In such meetings, nurses could collect events they have learned from, such as non-standard cases and how they solved them. They could do this by means of boards on which the events would be visible for as long as is needed for the whole team to read them (e.g. to compensate for shifts). In this way, sharing and learning becomes a team activity.

## 5 Discussion

In the present research, we investigated the teamwork dynamics of an orthopaedic team and identified points of intervention for improving teamwork in such teams. We carried out a qualitative study that included extensive observations of and interviews with 26 members of an orthopaedic team at a top clinical teaching hospital in the Netherlands. The analysis of the collected data allowed us to identify five themes that we translated into four design opportunities for intervention, namely: a) Improve daily rounds by reducing cognitive overload and promoting confidence; b) Improve collaboration by building empathy; c) Connect the patient with the professional team; and d) Support changes by fostering learning. The previous section described these four opportunities for teamwork intervention in orthopaedics. The following is a discussion of these opportunities in relation to their potential and challenges based on literature, beyond orthopaedics. We also discuss the theoretical basis to take into consideration when designing interventions for each opportunity.

### 5.1 Reflecting on design opportunities

#### 5.1.1 Improve daily rounds by reducing cognitive overload and promoting confidence

Our results showed the presence of hierarchical barriers, communication difficulties and multitasking, which resulted in cognitive overload. These findings are in line with previous literature that describes how power distance is detrimental to team collaboration and communication [1–3, 5, 24] and how multitasking and information overload make it more difficult to create a shared understanding [9, 24]. As a result, the first opportunity for intervention offers the potential to improve communication, increase team members’ confidence and ease cognitive load.

To effectively design in this direction, a few aspects need to be taken into account. First, it is necessary to understand how professionals are trained to communicate in different ways. In the specific case of doctors and nurses, studies show that doctors tend to convey (and need) concise information, whereas nurses are trained to ‘paint the big picture’ and thus have a narrative style of communication [2, 5, 24]. Second, studies suggest that to provide optimal care, it is necessary to transform a consolidated culture into a more equal relationship that is based on mutual trust and confidence [1, 5].

We argue that the way tools support an individual’s ability to create an understanding of the situation can increase the individual’s confidence and consequently support proactive behaviour and advocacy. Methods have been developed and validated that achieve the aforementioned goals, such as SBAR (Situation, Background, Assessment and Recommendation) [2, 24]. This tool provides a framework to structure the communication within professionals and helps those who use it to develop clinical thinking by formulating an assessment. In the same way that the SBAR method guides a conversation with an abstract format, interfaces can guide an exchange of information with visual cues. Eventually, solutions should be designed by taking into account that a great part of the work of a medical team is communicating and transferring information amongst different professional cultures. Therefore, systems should make the professionals read from the same page (literally and/or metaphorically) to reach an actual shared understanding [10].

#### 5.1.2 Improve collaboration by building empathy

Our findings underline the importance of creating a reciprocal understanding to achieve an effortless collaboration. This is supported by teamwork literature, where being aware of each other’s roles fosters the creation of a shared understanding, which is one of the most important factors for empowering teamwork [4, 5]. In healthcare, inter-professional training programmes are being promoted, since inter-professional team collaboration improves patient outcomes [25]. Such programmes focus on shared learning models, whereby students from different disciplines learn interdependently, through interaction, resulting in them valuing each other’s roles and approaches to patient care [25].

In line with the need to create opportunities for understanding colleagues’ work, we proposed solutions such as shadowing activities that aim at increasing empathy for each other’s needs. A similar solution has been developed by the United States Agency for Healthcare Research and Quality (AHRQ) [26], in the form of a checklist that can be used by team members who shadow a colleague and note down observations, guided by such questions as “Were any health care providers difficult to approach?”, to be answered in yes / no / n/a answer columns. Although a valuable example, we suggest that the format should be designed to elicit elaborated observations, rather than simple yes/no answers. The idea is to make team members reflect on pre-existing frictions and to ask questions that encourage reflection on specific topics.

In this paper, we suggested asking residents to observe moments at which a nurse needs to call a doctor and what information she sought. The goal of this activity is to understand challenges and strengths, and how each team member can contribute to improving collaboration. This creation of empathy also leads to a better understanding of shared objectives. In our case, the introduction of new procedures and protocols were situations that stressed the need for goal alignment. Besides inter-professional contact, an analysis revealed how the use of a ‘daily goals’ checklist during the rounds in an ICU improved the team members’ understanding of the goals by explicitly declaring objectives [27].

Activities like shadowing are time consuming and require a high level of willingness on the part of the team members. Therefore, implementing them could be challenging. However, because the improved teamwork outcomes are noteworthy, leaders should promote the use of such techniques.

#### 5.1.3 Connect the patient with the professional team

The third opportunity concerned the possibility of improving teamwork by including the patient as part of the team itself. Although this does not directly address interactions among team members, it is aimed at streamlining these interactions through their common point: the patient. A study supports this view by suggesting the “patient-as-a-professional” role, which recognises the time and effort patients devote to managing their health, thus contributing to the professionals’ team [28] by, for example, learning about their conditions or seeking clinicians who fit their mind-set.

Studies have shown that involving patients in their own healthcare, supporting a shared decision-making process and considering them beyond their medical condition, proved to be a path towards better clinical and financial outcomes (e.g. reducing LoS) in orthopaedics and other specialities [28–30]. From a teamwork perspective, literature shows that patients who experience good team coordination are more satisfied with the care they receive [3]. Therefore, it can be inferred that making patients feel part of the team and having them contribute to its work and witness the professionals’ attention to their needs, are ways to increase patient satisfaction.

Nevertheless, it has to be considered that patients’ profiles range from those who actively seek information and take decisions, to those who are more passive and depend on their clinicians. Their willingness to engage in the various activities is also variable, as shown by both our results and other research [28]. So, to provide all patients with the benefits of patient-centred care, it is necessary to involve them in the way that suits them best [28], adapting the amount and quality of information to their preferences [31]. We suggested aids for shared decision-making and collection of information, where professionals are guided in communicating with the patients according to their profiles.

A challenge lies in fitting these aids into the discussions without increasing the time spent on consultations or paperwork [28, 30]. Moreover, patients who have gathered information prior to their visit often requires more of their physicians’ time, as they want their physicians to verify their knowledge [28]. In our analysis, we found that although caregivers had the same type of concerns, they appreciated it when patients made the effort to truly understand their situation. Interventions should hence be designed to keep time management a priority, for instance by helping patients to prepare for their consultations.

#### 5.1.4 Support changes by fostering learning

The impact of changes on team composition and consequent interactions were also outlined in our results. Here, the change was due to the merging of two wards and the resultant need for both old and new staff to adapt. It is not surprising that the decision to merge wards was taken, since managing beds in a flexible way has financial benefits [32]. However, it also has disadvantages such as the need for additional training for nurses [32] as well as adaptation time, as our results showed. Consequently, intervening in this direction supports the professionals in shifting to a new way of managing health.

To design for professionals’ adaptation to change, the process of change needs to be acknowledged. Fisher’s process of transition [33] shows that this mechanism often includes an initial phase of negative emotions that can lead to either withdrawal from the situation or a gradual acceptance. Within a team that is going through these steps, the team leader has a central role in identifying the past and the envisioned experience of the people involved in the change to support and guide them through the process. We add that sharing knowledge and fostering learning are ways to help team members in such transitions. To do so, the learning needs of each individual should be taken into account, in order to provide the correct level of engagement in the learning activities [34]. For example, for nurses who already have years of experience in a certain speciality, having to switch to a new one constitutes a radical change. However, they have extensive knowledge in a parallel sector and can thus contribute to teaching others.

We proposed the use of huddles for providing opportunities for shared learning and fostering knowledge dissemination, using boards that are visible to the whole staff. In previous research, another case is that of a multidisciplinary trauma round, in which participants were given different roles (such as ‘challenger’ or ‘inquisitor’) to structure the meeting in a way that would foster learning. By using this method, more learning-oriented, constructive discussions were achieved [34]. An important element in learning is to avoid judging one’s skills in order to create psychological safety [2]: team members need to be open to create a positive environment for learning, where everybody is in the process of learning something new. Even for this type of intervention, time management becomes a possible pitfall, which is why we suggested extending the scope of current briefing activities towards a more learning-oriented huddle.

### 5.2 Combining opportunities

Although the four opportunities we outlined are focussed on specific factors, they can be considered a holistic package to improve teamwork consistently over the long run. The identified directions share the goal of creating common knowledge, fostering learning and achieving mutual understanding. As a result, intervening in one opportunity influences the others as well, more or less directly. For example, fostering learning within the nursing team is a way for them to increase their confidence about their own knowledge, thus potentially making them more capable of showing proactive behaviour when discussing care plans with doctors. Increasing the understanding of each other’s job, for instance between nurses and doctors, should make their collaboration better on a broader level. Ensuring that objectives are understood by the whole team is reflected in the way patients experience the team, as their satisfaction increases when their interactions with each team member are coherent.

### 5.3 Limitations, contribution and further research

The results of our study are based on one hospital and the experiences of a small sample. Hence, although similar situations may exist in other similar contexts, the results cannot be generalised. Nevertheless, the insights we gained and the opportunities for intervention we proposed are in line with previous research. As compared to existing literature, we applied a design approach focusing on team members’ experiences, in an attempt to go beyond the theory and suggest concrete solutions for intervention. Employing this perspective helped us to obtain a holistic view of the team dynamics from the various stakeholders’ points of view. This factor is relevant since a key element for the introduction of new tools is to take into account and iteratively assess the different stakeholders’ needs, in order to avoid the manifestation of “stakeholders dissonance” once the tools are in place [35]. What we proposed is not an exhaustive list of solutions, but rather pointers for developing interventions.

Moreover, the solutions we discussed have not yet been tested, although they are based on elements of existing interventions. Future research should investigate the implementation of the interventions and their effect on teamwork dynamics within healthcare teams, not necessarily orthopaedics. It would be also valuable to focus on analysing the effect of the solutions’ combined action. We believe that the process we applied and the insights we gained can inspire designers who approach the topic of teamwork in healthcare, and provide hospital managers and team leaders with instruments to support and enhance teamwork.

## 6 Conclusions

This paper contributes to the discourse on teamwork in healthcare by providing an understanding of teamwork dynamics and related strengths and weaknesses within an orthopaedic team. The results offer insights for improvement for both the healthcare and the design community, by translating the identified dynamics into directions designers can concretely intervene upon. Moreover, the paper shows an example of a way to explore teamwork, taking into account behavioural aspects of the interaction among team members.

Although this study covered a sample of team members at one hospital, the results are in line with previous research on teamwork in healthcare, especially concerning the importance of creating a shared understanding. Specific contextual differences have to be taken into consideration, but future work can make use of the provided insights to design and evaluate tools and products aimed at facilitating teamwork in orthopaedics and other healthcare domains.

## Conflict of interest

The authors have no conflict of interest to report.
